# Adrenomedullin Secreted by Melanoma Cells Promotes Melanoma Tumor Growth through Angiogenesis and Lymphangiogenesis

**DOI:** 10.3390/cancers14235909

**Published:** 2022-11-29

**Authors:** Zohra Benyahia, Caroline Gaudy-Marqueste, Caroline Berenguer-Daizé, Norhimane Chabane, Nadège Dussault, Mylène Cayol, Christine Vellutini, Amina Djemli, Isabelle Nanni, Nathalie Beaufils, Kamel Mabrouk, Jean-Jacques Grob, L’Houcine Ouafik

**Affiliations:** 1 Aix Marseille Univ, CNRS, INP, Inst Neurophysiopathol, 13005 Marseille, France; 2 Aix Marseille Univ, APHM, CHU Timone, Service de Dermatologie et de Cancérologie Cutanée, 13005 Marseille, France; 3 Aix Marseille Univ, APHM, CHU Nord, Service D’anatomopathologie, 13015 Marseille, France; 4 Aix Marseille Univ, APHM, CHU Nord, Service D’Onco-Biologie, 13015 Marseille, France; 5 Aix Marseille Univ, CNRS, ICR, Institut de Chimie Radicalaire, 13013 Marseille, France

**Keywords:** adrenomedullin, melanoma, tumor growth, angiogenesis, lymphangiogenesis, invasion, A375, MeWo, SK-MEL-28

## Abstract

**Simple Summary:**

Adrenomedullin (AM) and AM receptors were immunohistochemically localized in the primitive and metastatic melanoma specimens, suggesting a role of the adrenomedullin system in melanoma growth. Adrenomedullin functions as an autocrine/paracrine growth factor to stimulate proliferation, migration, and invasion of A375, MeWo, and SK-MEL-28 cells, whose effect is inhibited by neutralizing anti-AM and anti-AM receptor antibodies, causing cessation of growth, migration, and invasion in vitro. The in vivo study highlights the significance of adrenomedullin as an important factor that promotes melanoma tumor growth and affects the tumor microenvironment by inducing pathologic neoangiogenesis and lymphangiogenesis. Targeting the adrenomedullin system may provide a rational basis for future therapeutic modalities in melanoma.

**Abstract:**

Introduction: Metastatic melanoma is an aggressive tumor and can constitute a real therapeutic challenge despite the significant progress achieved with targeted therapies and immunotherapies, thus highlighting the need for the identification of new therapeutic targets. Adrenomedullin (AM) is a peptide with significant expression in multiple types of tumors and is multifunctional. AM impacts angiogenesis and tumor growth and binds to calcitonin receptor-like receptor/receptor activity-modifying protein 2 or 3 (CLR/RAMP2; CLR/RAMP3). Methods: In vitro and in vivo studies were performed to determine the functional role of AM in melanoma growth and tumor-associated angiogenesis and lymphangiogenesis. Results: In this study, AM and AM receptors were immunohistochemically localized in the tumoral compartment of melanoma tissue, suggesting that the AM system plays a role in melanoma growth. We used A375, SK-MEL-28, and MeWo cells, for which we demonstrate an expression of AM and its receptors; hypoxia induces the expression of AM in melanoma cells. The proliferation of A375 and SK-MEL-28 cells is decreased by anti-AM antibody (αAM) and anti-AMR antibodies (αAMR), supporting the fact that AM may function as a potent autocrine/paracrine growth factor for melanoma cells. Furthermore, migration and invasion of melanoma cells increased after treatment with AM and decreased after treatment with αAMR, thus indicating that melanoma cells are regulated by AM. Systemic administration of αAMR reduced neovascularization of in vivo Matrigel plugs containing melanoma cells, as demonstrated by reduced numbers of vessel structures, which suggests that AM is one of the melanoma cells-derived factors responsible for endothelial cell-like and pericyte recruitment in the construction of neovascularization. In vivo, αAMR therapy blocked angiogenesis and lymphangiogenesis and decreased proliferation in MeWo xenografts, thereby resulting in tumor regression. Histological examination of αAMR-treated tumors showed evidence of the disruption of tumor vascularity, with depletion of vascular endothelial cells and a significant decrease in lymphatic endothelial cells. Conclusions: The expression of AM by melanoma cells promotes tumor growth and neovascularization by supplying/amplifying signals for neoangiogenesis and lymphangiogenesis.

## 1. Introduction

Melanoma consists of a heterogeneous group of tumor cells that vary greatly in their malignant potential. When excised early, primary melanoma usually has an excellent prognosis. At a given point, primary melanomas gain the ability to cross the basal layer of the epidermis and invade the deeper dermal layers of the skin, which confers them the potential to metastasize. Among different events characterizing this phase in primary melanoma, the angiogenic switch describing the ability to induce numerous pro-angiogenic factors is probably an important step. The pro-angiogenic features are even more enhanced in melanoma metastases [1]. The potential efficacy of antiangiogenic therapy is, thus, important, although few clinical data have confirmed this concept.

Tumor growth may be simultaneously driven by a combination of autocrine and paracrine mechanisms through the production of growth factors and expression of their cognate receptors [2]. One of the genes implicated in these processes is the adrenomedullin (AM) gene, or *ADM*; its expression is involved in the normal functioning of various cell types (e.g., bone marrow stromal cells and endothelial cells), but also in numerous lines of tumor cells [3,4,5]. Some of the key functional properties of AM include angiogenesis, regulation of cellular growth, and induction of vasodilation [3,6,7]. This peptide functions by binding to the calcitonin receptor-like receptor (CLR), which is a type of G protein-coupled receptor, specifically in association with receptor activity-modifying proteins 2 (RAMP2) and 3 (RAMP3) [8]. It is inferred from the high responsiveness of both CLR/RAMP2 and CRLR/RAMP3 to stimulation by AM that there are also two corresponding AM receptors, designated AMR_1_ and AMR_2_ [9].

Despite its importance for cell functioning, AM and the AMR receptors have also been demonstrated to play a role in the development of multiple types of tumors [10,11]. It has been reported that AM and its receptors are present in all the epithelial cells in human skin, the normal tissue from which melanomas arise [12]. The expression has been reported in keratinocytes of the epidermis and their follicles, as well as cells of the glands and secretory ducts [12]. In addition, AM and its receptors were found in skin tumors of different histologies [12]. Previous work has reported that a major source of AM in melanoma is tumor-associated macrophages [13]. Higher levels of AM expression have also been found to be associated with more rapid progression and earlier mortality for some types of cancer [5,14,15]. These outcomes can likely be linked to findings that AM plays a role in both proliferation and the inhibition of apoptosis in many types of cells [6,16,17,18], including multiple forms of malignancy [5,14,19,20,21]. AM is also thought to contribute to the growth of tumors by promoting lymphangiogenesis and angiogenesis [19,20,21,22,23,24]. Previous research has supported this hypothesis by demonstrating that using the antagonistic peptide AM_22–52_ [25], which interferes directly with AM receptors [26], and employing antibodies that work to neutralize either AM (αAM) or its receptors can effectively reduce tumor proliferation in vitro as well as inhibit the growth of experimental tumors in vivo [20,21].

Thus, the AM/AM receptor pathway appears to be a potential target for the development of therapies aimed at the treatment of melanoma. The aim of the present study was to explore important elements of the system comprising AM and its receptors, because its expression and function has not yet been fully described. To do so, we performed a combination of in vitro and in vivo tests, examining the role of the AM system on the growth of melanoma cell lines and xenografts, respectively.

## 2. Materials and Methods

### 2.1. Human Melanoma Tissues

Specimens of human melanoma were obtained from 4 patients with either primitive melanoma (n = 1) characterized by cKit insertion (p. Trp557_Lys558insLys) (patient n°4) or metastatic melanoma (n = 3) characterized by N-Ras mutation (Q61L) (patient n°1; hepatic metastatic melanoma); B-RAF mutation (V600E) (patient n°2; grele metastatic melanoma); or B-RAF mutation (V600E) (patient n°3; metastatic melanoma). After patients provided consent, paraffin-embedded specimens were provided by the AP-HM Tumor Tissue Bank (AC-2013-1786). Specimens were de-identified according to protocols approved by the appropriate ethical review committees (APHM/Aix-Marseille University). Samples were analyzed for the presence of AM, CLR, RAMP2, and RAMP3 proteins using a methodology that has been previously described [14,22].

### 2.2. Immunohistochemistry of the AM System in Human Melanoma

Sections of paraffin-embedded samples (6 μm) of human melanoma cancer specimens were analyzed for AM, CLR, RAMP2, and RAMP3 proteins as previously described [14,22], and protein staining was evaluated by an experienced pathologist. Immunohistochemistry was performed using the Vectastain Elite ABC Universal Kit (Vector laboratories, Burlington, CA, USA). Optimal dilutions for anti-AM and anti-CLR antibodies were 1/1500; anti-RAMP2 antibody was 1/1000; and anti-RAMP3 antibody was 1/750. Detection was performed using a diaminobenzedine chromogen, which resulted in a positive brown staining. Sections were counterstained with hematoxylin, dehydrated in ethanol, and mounted with glass coverslips. As a control for immunostaining, antibodies that had been preadsorbed by human synthetic AM peptide (50 μM; Bachem, Bubendorf, Switzerland), CLR, RAMP2, and RAMP3 peptides (50 μM, CROPS laboratory, CNRS) were used instead of primary antibodies.

### 2.3. Cell Lines and Hypoxic Treatment

Melanoma cell lines A375 and SK-MEL-28 originated from primitive melanoma and MeWo originated from metastatic melanoma; cell lines were obtained from the American Type Culture Collection (ATCC, Rockville, MD, USA). Cells were grown in a humidified atmosphere at 37 °C in 95% air, 5% CO_2_ in DMEM (Lonza BioWhitaker, Illkirch-Graffenstaden, France) for A375 and SK-MEL-28 cells and MEM (Lonza BioWhitaker) for MeWo cells supplemented with L-glutamine (2 mM) and 10% heat-inactivated Fetal Bovine Serum (FBS) for normoxic conditions. The induction of hypoxia was achieved by using 260 μM of the hypoxia mimetic desferrioxamine mesylate (DFX) (Sigma, Paris, France). After cells had grown to a confluence of 70%, the medium was changed and cells were incubated with new medium containing 260 μM DFX for 24 and 48 h.

### 2.4. RNA Preparation and Real-Time Quantitative RT-PCR

Preparation of total RNA was carried out using A375, SK-MEL-28, and MeWo cells. Reverse transcription to cDNA was then conducted using a methodology described previously [27]. The LC480 PCR system (Roche Diagnostics, Meylan, France) was used for reverse transcription of human AM, CLR, RAMP2, RAMP3, and GAPDH mRNA using a previously established methodology [27].

### 2.5. Immunostaining of the Melanoma Cells

The fluorescence microscopy analyses of AM, CLR, RAMP2, and RAMP3 were performed on the A375, SK-MEL-28, and MeWo cells as described [28]. Briefly, after cells were fixed in 4% paraformaldehyde and permeabilized with 0.1% Triton X-100, cells were incubated with polyclonal antibodies against AM (1:2000), CLR (1:2000), RAMP2 (1:1500), and RAMP3 (1:1000) overnight at 4 °C, and then washed and incubated with Alexa Fluor-conjugated antibodies (1:300; Vector Laboratories, Burlingame, CA, USA) for 45 min at room temperature (RT). After washing, the samples were mounted in VETASHIELD (Vector Laboratories) and analyzed by fluorescence microscopy.

### 2.6. Cell Proliferation Assay

Cell lines A375 (1 × 10^3^ cells), SK-MEL-28 (2 × 10^3^ cells), and MeWo (4 × 10^3^ cells) were seeded in 24 multiwells. The effects of AM (10^−7^ M), rabbit anti-human AM (αAM; 70 μg/mL), and anti-human AMR (αCLR, αRAMP2, αRAMP3; αAMRs; 70 μg/mL)-neutralizing antibodies (purified IgG) developed in-house [3,20,21] or non-immune purified IgG (70 μg/mL) were added daily to the culture to evaluate their effects on cell proliferation. After six days of treatment in six wells treated with AM, αAMR, or purified IgG, the effects were examined using a 3-(4,5-dimethylthiazol-2, 5-diphenyltetrazolium bromide) (MTT) assay (Promega, Lyon, France). The Bio-Tek microplate was used to determine the change in the number of viable cells from dye reduction measured by absorbance at 570 nm. The values represent the mean ± SD of five independent experiments with six wells each.

### 2.7. Cell Migration and Invasion Assays

In order to examine chemoinvasion and migration of A375 cells (20 × 10^3^), SK-MEL-28 cells (20 × 10^3^), MeWo cells (1 × 10^5^), and the murine bone marrow-derived cells (BMDCs, 5 × 10^5^), we used a modified Boyden chamber assay, as described previously [3,12,26]. Briefly, for chemoinvasion, the filter was coated with a layer of Matrigel (0.5 mg/mL, Becton Dickinson, Paris, France). A375, SK-MEL-28, and MeWo cells were harvested by trypsinization, collected by centrifugation and resuspended in DMEM (A375 and SK-MEL-28 cells) and MEM (MeWo cells) containing 0.5% bovine serum albumin (BSA) and soybean trypsin inhibitor, washed 3 times with the medium containing BSA as above, and suspended in the same medium at a concentration of 2 × 10^5^ cells/mL for A375 and SK-MEL-28 cells and 1 × 10^6^ cells/mL for MeWo cells. The bone marrow-derived cells (BMDCs) were prepared in DMEM medium at 5 × 10^6^ cells/mL. A total of 100 μL of this suspension was added to the upper compartment (24-multiwell chemotaxis Boyden microchamber). The lower compartment of the chamber was filled with chemoattractant AM diluted in DMEM (A375, SK-MEL-28 cells, and BMDCs) or in MEM (MeWo cells) (n = 4 in triplicate). Where indicated, cells were pre-incubated for 30 min at 37 °C with αAMRs or with preimmune serum (affinity-purified IgG). After incubation at 37 °C for 4 hr, cells that had not migrated from the top of the filter were scraped away with a cotton applicator. The filter was fixed for 30 min at RT with 3.7% paraformaldehyde, washed two times with PBS, and stained with 4′,6′-diamidino-2-phenylindole (DAPI). The number of cells that migrated to the lower surface of each membrane was counted at 50X magnification using a microscope. The control well was filled with DMEM or MEM containing 2% FBS. Data are expressed as the number of migrated cells in 10 high-power fields, and the values represent the mean ± SD of four independent experiments, each performed in triplicate.

### 2.8. In Vivo Matrigel Plug Studies

C57BL/6 female mice were injected subcutaneously above the rectus abdominus with 0.8 mL of Matrigel (Becton Dickinson, Le Pont de Claix, France) admixed to A375 cells (1 × 10^6^) (n = 5), SK-MEL-28 cells (1.5 × 10^6^) (n = 5), or MeWo cells (2 × 10^6^) (n = 5) in 50 μL of PBS or alone as a negative control (n = 5). Twenty-four hours later, each group of mice was randomized into two groups and treated intraperitoneally with αAMRs (12 mg/kg) or preimmune serum (purified IgG, 12 mg/kg) every three days. Three weeks later, animals were euthanized, and the Matrigel plugs were dissected and fixed in 4% paraformaldehyde (PFA) for histological analysis. Immunohistochemistry was performed on paraffin-embedded sections using the Vectastatin Elite ABC Universal kit (Vector Laboratories, Burlingame, CA) as described previously [14,22]. Antibodies recognizing CD31 (1:20; Dianova, Geneva, Switzerland) and lymphatic vessel hyaluronic acid receptor-1 (LYVE-1; 1:100) (Dako Inc., Glostrup, Denmark) were used for analysis. For each marker, whole-surface staining was quantified using Image J software (NIH, Bethesda, MD, USA). CD31- and LYVE-1-positive cells are shown; they were analyzed based on 5 magnification fields (400×) per section. Immunohistochemical staining of the endothelial cell surface marker CD31 was used to determine microvessel density. The blood vessels were counted randomly from non-necrotic areas in each Matrigel section using a ×200 microscope field on CD31-stained Matrigel sections. Quantitative assessment of the density of cells that stained positive for CD31 or LYVE-1 was conducted for the entire surface of the corresponding slides using CALOPIX software (n = 6 per animal, total number of animals (n = 5)). MBF_ImageJ 1.52a Software was used for the analysis.

### 2.9. In Vivo Tumor Growth

In vivo tumor growth was assessed in 20 female athymic naval medical research institute (NMRI; nu/nu) nude mice (Harlan Laboratories SARL, Gannat, France) purchased at 5 weeks of age who had been injected with suspensions of MeWo cells (2 × 10^6^ in 100 μL PBS) subcutaneously into the right flank. Dial-caliper measurements were taken to determine tumor size and volume (volume = width × length × height × 0.5236). After tumor growth had reached a volume of 250 ± 50 mm^3^, animals were randomly assigned to treatment or control groups. The treatment group (n = 10) was given an intraperitoneal (i.p.) injection of αAMRs (12 mg/kg purified IgG in 200 μL PBS) every 3 days, as described in a previous methodology [21]. The control group (n = 10) was given an i.p. injection of an irrelevant antibody (IgG of the same isotype). All IgG preparations were tested for endotoxin using the Pyrogent plus Limulus amebocyte lysate kit (Lonza). All antibody preparations used in animal studies contained less than <1.25 U/mL endotoxin. Tumors sizes were measured every 3 days and mice were euthanized 9 weeks after injection. If the size of subcutaneous (s.c.) tumors reached 1800 mm^3^, then the animal was humanely killed according to the guidelines established by the Aix-Marseilles University Animal Rights Committee. Tumors were embedded in paraffin for pathologic studies and immunohistochemistry.

### 2.10. Immunohistochemical Staining

Formalin-fixed paraffin-embedded xenografts were cut into 6 μm thick sections, and immunochemical analysis was conducted using the Vectastain Elite ABC Universal kit (Vector Laboratories, Burlingame, CA, USA) using a previously described methodology [14,22]. Antibodies against CD31 (1:20, Dianova, Geneva, Switzerland), lymphatic vessel hyaluronic acid receptor-1 (LYVE-1) (1:100; DAKO, Glostrup, Denmark), and Ki-67 nuclear antigen (1:100; Dako, Glostrup, Denmark) were used for the analysis. For each marker, whole-surface staining was quantified using Image J software (NIH, Bethesda, MD, USA).

### 2.11. Statistical Analysis 

For statistical analysis, non-parametric Kruskal–Wallis analysis followed by the Bonferroni test was performed using XLSTAT Software (XLSTAT BASIC; Addinsoft, Paris, France) throughout the whole manuscript. Results in bar graphs are given as mean values and their corresponding standard deviation (SD). For all tests, differences were considered statistically significant when *p* < 0.05.

## 3. Results

### 3.1. Immunohistochemistry of AM, CLR, RAMP2, and RAMP3 Proteins in Human Melanoma

**Figure 1:**
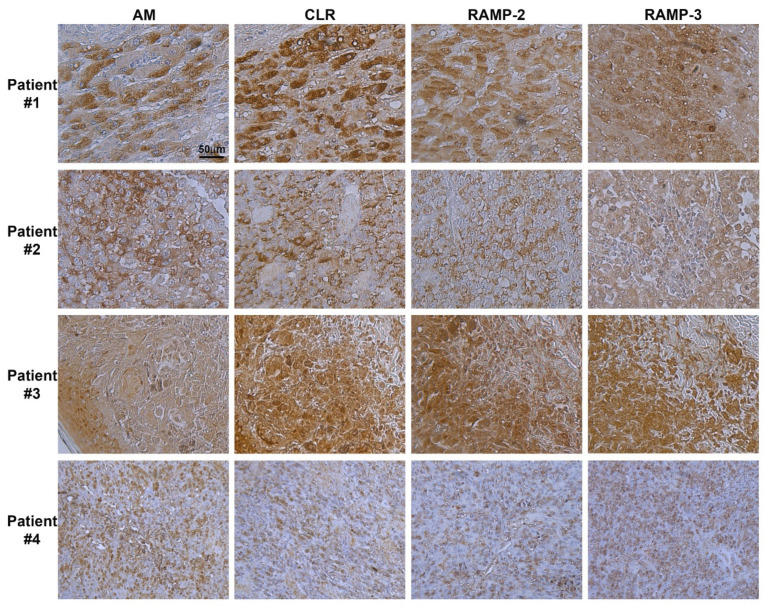
Expression of AM and its receptors in human melanoma. Immunohistochemistry for AM, CLR, RAMP2, and RAMP3 in melanoma tissue. Strong cytoplasmic staining for AM, CLR, RAMP2, and RAMP3 is observed in melanoma cells. Stroma cells with weaker cytoplasmic staining are also observed.

Antibodies were used to label serial sections of melanoma tissue with corresponding AM, CLR, RAMP2, and RAMP3 proteins (Figure 1). Within all tumor cells, the immunostaining procedure revealed the presence of AM, CLR, RAMP2, and RAMP3, as depicted in Figure 1. Melanoma cells were also strongly labelled for AM, CLR, RAMP2, and RAMP3 in melanoma metastatic tissue (Figure 1; patients #1, 2, 3), while mild staining can be observed in melanoma primitive tissue (Figure 1; patient #4). Patient #1 presents an NRAS^Q61L^ mutation, patients #2 and #3 both demonstrate a BRAF^V600E^ mutation, and patient #4 showed a c-Kit^W557_k558insk^ insertion. Whether the increased staining of AM, CLR, RAMP2, and RAMP3 in patients #1, #2, and #3 versus patient #4 is due to these specific mutations needs further investigation. Positive staining for AM, CLR, RAMP2, and RAMP3 disappeared entirely when the antibodies were preabsorbed with 50 μM of synthetic AM, CLR, RAMP2, and RAMP3 peptides (Supplementary Materials Figure S1). Interpreted as a whole, these findings suggest that the AM system is well expressed in melanoma tissue and may be involved in the growth of tumor cells in both in vitro and in vivo settings.

### 3.2. Expression of AM and AM Receptors in Melanoma Cells

**Figure 2:**
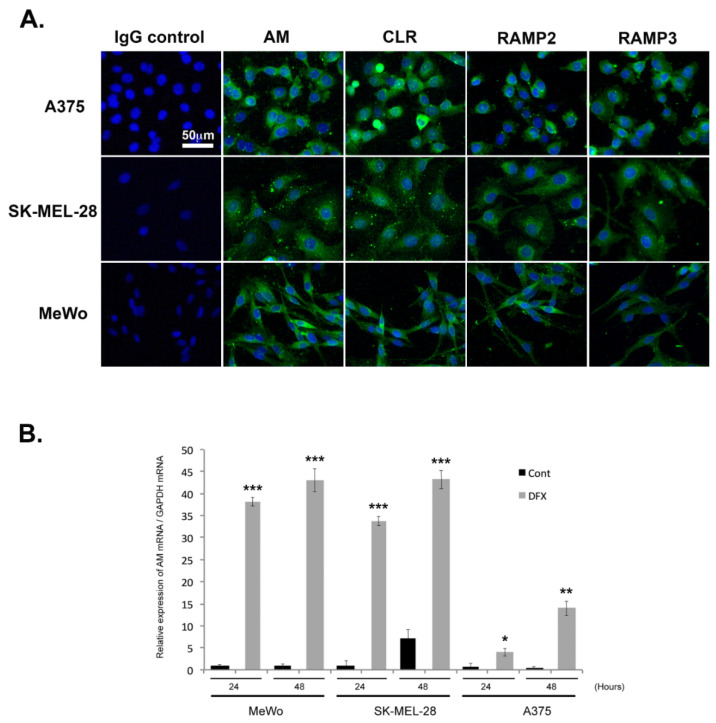
Depiction of the extent to which AM signaling is expressed and regulated in melanoma cells. (**A**) AM and receptors expressed in melanoma cells depicted using immunofluorescence of A375, SK-MEL-28, and MeWo cells stained with antibodies against AM, CLR, RAMP2, and RAMP3, revealing localization in the cytoplasm. Negative control for immunostaining was achieved with IgG-control. (**B**) AM expression induced by a hypoxia mimetic in melanoma cells. Total RNA (1 μg, DNA free) prepared from MeWo, SK-MEL-28, and A375 cells were reverse transcribed into cDNA under normoxic or hypoxic conditions and relative AM mRNA was estimated using a real-time quantitative reverse transcriptase polymerase chain reaction. There were significant differences between cells treated with desferrioxamine mesylate (DFX) and untreated control cells in terms of AM expression: * *p* < 0.05; ** *p* < 0.01; *** *p* < 0.001. Each experiment is representative of five independent experiments. Results are shown as means ± SD.

The observation of a clear pattern of expression of AM and AM receptors in the tumor samples examined here supports the hypothesis that this system is implicated in melanoma formation and progression. The approach taken here was to use A375, SK-MEL-28, and MeWo cells to help understand how the AM system functions within melanoma cells. We examined the presence of AM and AM receptors, as well as their localization within cells under normoxic conditions, using immunofluorescence. Representative example images are presented in Figure 2A, with A375, SK-MEL-28, and MeWo cells having been immunostained for AM, CLR, RAMP2, and RAMP3. Given these normoxic conditions, AM, CLR, RAMP2, and RAMP3 staining generally remained localized to the cytoplasm (Figure 2A). Positive staining was completely abolished by pre-absorption of the antibody with 50 μM of synthetic peptide (not shown). Staining in the presence of IgG rabbit was negative for all melanoma cells (Figure 2A).

### 3.3. Regulation of AM Expression by Hypoxia

Quantitative reverse transcriptase PCR (RT-PCR) analysis demonstrated that A375, SK-MEL-28, and MeWo cells express AM mRNA (Figure 2B). Under hypoxic conditions, the levels of AM mRNA increased 38- and 43-fold in MeWo cells (Figure 2B), 33- and 48-fold in SK-MEL-28 cells (Figure 2B), and 5- and 23-fold in A375 cells (Figure 2B) after treatment for 24 h and 48 h, respectively. No increased expression could be observed for CLR, RAMP2, or RAMP3 mRNAs under hypoxia (Supplementary Materials Figure S2).

### 3.4. Effects of AM and AM Blockade on Melanoma Cell Proliferation

**Figure 3:**
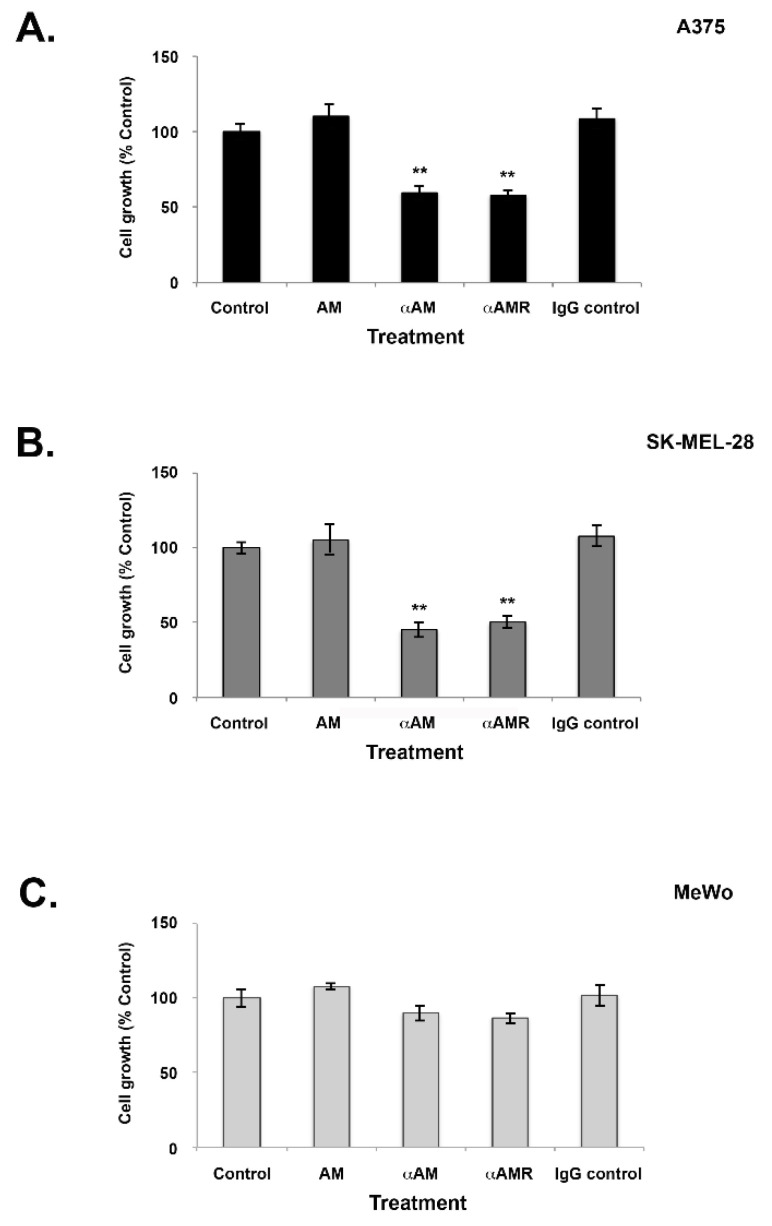
Effect of AM on in vitro growth of melanoma cells. (**A**–**C**) Cells were seeded at densities of 1 × 10^3^ (A375), 2 × 10^3^ (SK-MEL-28), and 4 × 10^3^ (MeWo) per well for the proliferation assay in 24 multiwell plates using a growth medium containing 2% of fetal bovine serum. AM (10^−7^ M), αAM (70 μg/mL), αAMRs (70 μg/mL), or control IgG (70 μg/mL) was added to the cells for 6 days of treatment. Six wells were prepared for each treatment for 3-(4,5-dimethylthiazol-2-yl)-2,5-diphenyltetrazolium bromide (MTT) analysis. ** *p* < 0.01. The values represent the mean ± SD of five independent experiments, each performed in triplicate.

The observation that AM and its receptors are expressed in melanoma tissues and cell lines provides evidence that the AM system may be implicated in the growth of melanoma cells due to the growth loop involving the autocrine and paracrine systems. After 6 days of treatment with AM (10^−7^ M), none of the three melanoma cell lines (A375, SK-MEL-28, or MeWo) demonstrated increased proliferation compared to controls (Figure 3). Conversely, when treated with αAM- or αAMR-neutralizing antibodies, cell proliferation was reduced by up to 30% for the A375 line (*p* < 0.001) and by 40% to 50% for the SK-MEL-28 line (*p* < 0.001) compared to controls (Figure 3A,B). These observations are consistent with action via autocrine functionality and indicate that the involvement of AM in the growth of melanoma cell lines A375 and SK-MEL-28 is mediated through the AMR_1_ and/or AMR_2_ receptors. MeWo cells did not show any decrease in proliferation after treatment with either αAM- or αAMR antibodies despite the expression of CLR, RAMP2, and RAMP3, suggesting that AM is not involved in MeWo cell growth in vitro (Figure 3C).

### 3.5. AM Induces Melanoma Cell Migration and Invasion In Vitro

**Figure 4:**
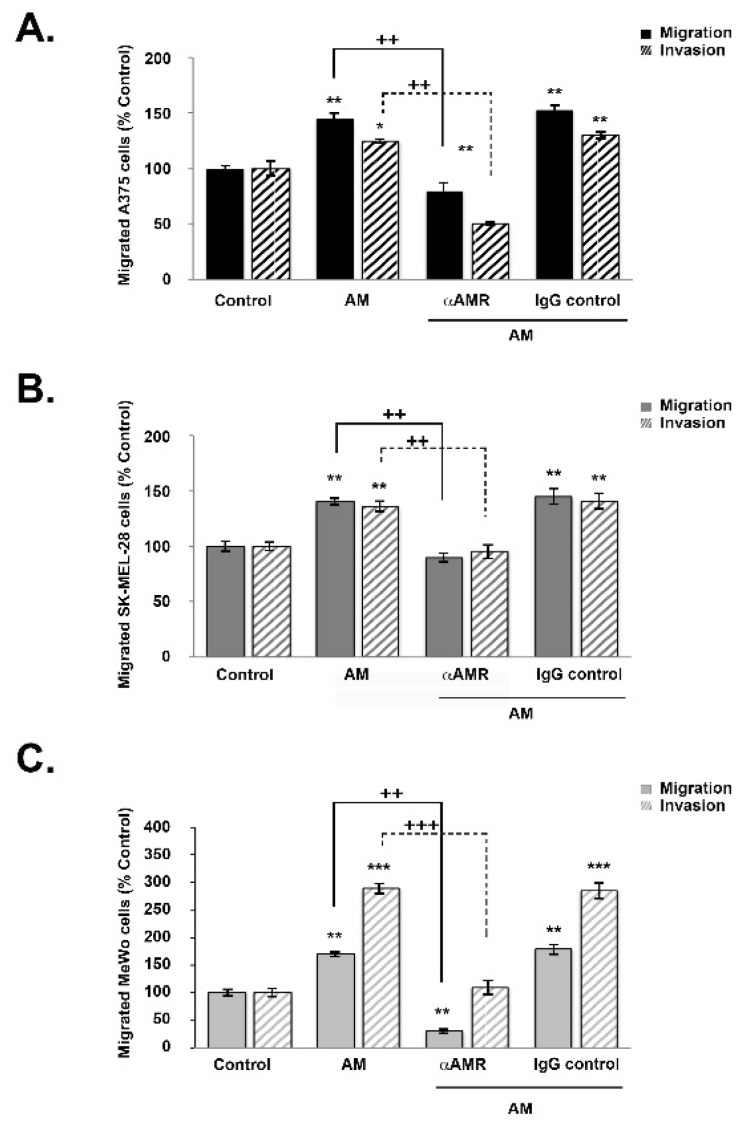
AM regulates melanoma cell migration and invasion in vitro. (**A**–**C**) The bottom wells of all chambers were filled with DMEM for A375 and SK-MEL-28 cells or MEM for MeWo cells containing 2% fetal bovine serum in the presence of control buffer (control) or AM (10^−7^ M). A375 ((**A**), 2 × 10^4^ cells), SK-MEL-28 ((**B**), 2 × 10^4^ cells), or MeWo ((**C**), 1 × 10^5^ cells) cells pretreated for 30 min with αAMR (70 μg/mL) or control IgG (70 μg/mL) were placed in the upper chamber and incubated for 16 h at 37 °C. The cells that migrated were stained with 4′, 6′-diamidino-2-phenylindole and counted at 50x magnification using a microscope. Data are expressed as the number of migrated cells in 10 high-power fields, and the values represent the mean ± SD of four independent experiments, each performed in triplicate. The asterisk (*) is used for comparison to control cells (* *p* < 0.05; ** *p* < 0.01; *** *p* < 0.001) and the plus symbol (+) is used for comparison to AM-treated cells (++ *p* < 0.01; +++ *p* < 0.001).

To examine the extent to which AM influences melanoma cell motility, cells from each line (A375, SK-MEL-28, and MeWo) were incubated in a Boyden chamber assay for periods of 4, 16, and 24 h while exposed to AM. For each period and condition, the number of cells that had moved to the lower surface of the transwell was measured (Figure 4). There was a significant increase in migration and invasion in the presence of AM (10^−7^ M) for all three cell lines (*p* < 0.01) (Figure 4). The greatest magnitude of increase was observed for invasion among MeWo cells (*p* < 0.001) (Figure 4C). Induction of migration and invasion by AM was significantly inhibited when the melanoma cells were pre-incubated for 30 min with αAMRs (Figure 4). This overall pattern held for all three cell lines; however, invasion was more strongly inhibited for A375 cells (*p* < 0.01), while migration was more strongly inhibited among MeWo cells (*p* < 0.01) (Figure 4A and C, respectively). These results appear to indicate that cell invasion is promoted by AMR_1_ and/or AMR_2_. Collectively, these results suggest the existence of an autocrine loop involving AM receptors and AM secretion, which impacts the rate of migration and invasion in melanoma cells (Figure 4). Conversely, preimmune control IgG (70 μg/mL) had no significant effect on the way in which AM acted to stimulate migration or invasion in melanoma cell lines (Figure 4).

### 3.6. AM Released by Melanoma Cells Contributes to Angiogenesis and Lymphangiogenesis in Matrigel Plug Bioassays

**Figure 5:**
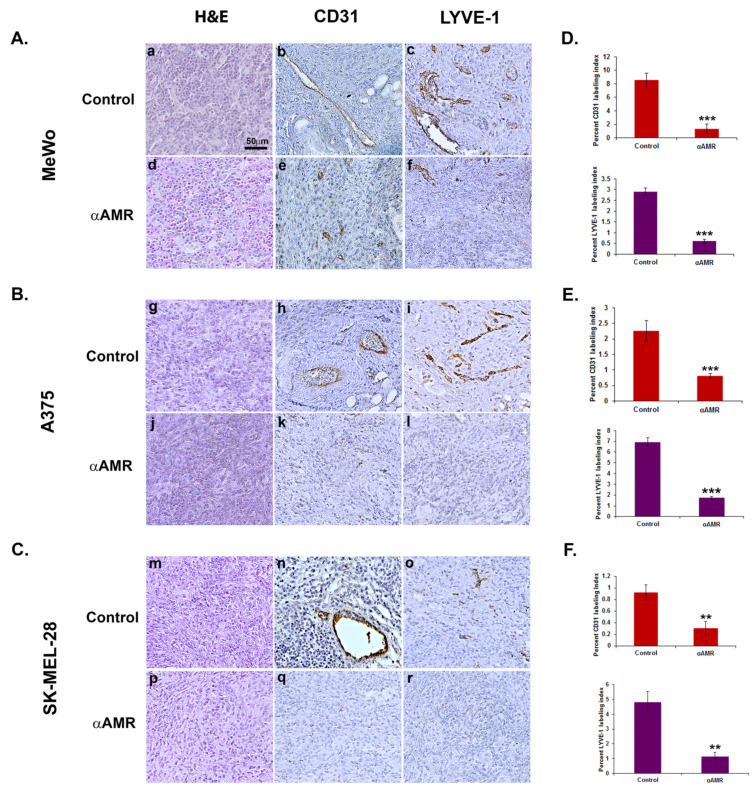
Analysis of in vivo Matrigel plug bioassays indicates that AM secreted by melanoma cells induces angiogenesis and lymphangiogenesis. (**A**–**C**) A total of 0.8 mL of growth factor-depleted Matrigel was admixed to MeWo ((**A**), 1 × 10^6^ cells) (**a**,**b**,**c**,**d**,**e**,**f**), A375 ((**B**), 1.5 × 10^6^ cells), or SK-MEL-28 ((**C**), 2 × 10^6^ cells) cells and administered to C57BL/6 mice via s.c. injection at the abdominal midline. Administration of αAMRs or control IgG was conducted intraperitoneally every three days (starting 24 h after initial Matrigel injection and for 15 days thereafter) in C57BL/6 mice. Formalin was used to fix Matrigel plugs, which were then embedded, sectioned, and used for immunohistochemical analysis. Figure 5A–C depict microphotographs of histochemical-stained Matrigel sections for H & E (**a**,**d**,**g**,**j**,**m**,**p**), blood vessel staining with the CD-31 antibody (**b**,**e**,**h**,**k**,**n**,**q**), and lymphatic vessels with the anti-LYVE-1 antibody (**c**,**f**,**i**,**l**,**o**,**r**) derived from Matrigel plugs mixed with melanoma cells treated with either αAMRs or control IgG. Each panel represents multiple fields, including five plugs in each group. Scale bar, 50 μm. (**D**–**F**) Quantitative assessment of cell density for CD31- and LYVE-1-positive cells as assessed by staining conducted on the entire surface of the corresponding slides using CALOPIX software. (v2.10.16 by Tribvn) MBF_Image J 1.52a software was used for the analysis. The values represent the means ± SD (** *p* < 0.01; *** *p* < 0.001).

It was further hypothesized that AM may also be implicated in angiogenic activity in melanomas. To test this hypothesis, we used in vivo Matrigel plug bioassays to measure lymphangiogenesis and angiogenesis in response to AM secretion in a non-inflammatory setting. To demonstrate that AM secreted by melanoma cells is involved in the promotion of the vascular and lymphatic channels, we used treatment with αAMRs to inhibit recruitment of circulating AMR-positive cells, as reported previously [21]. Matrigel plugs supplemented with A375, SK-MEL-28, or Mewo cells were injected subcutaneously into C57BL/6 mice, forming semisolid plugs. Twenty-four hours later, mice were treated by intraperitoneal (i.p.) injection with αAMRs or control-IgG at a dose of 350 μg every 3 days for a total of 15 days. The Matrigel plug bioassays conducted in vivo to assess angiogenesis indicated that plugs mixed with melanoma cells injected in animals that received control-IgG injections were well vascularized (Figure 5). Immunostaining using the vascular endothelial cell marker (CD31) or lymphatic endothelial cell marker (LYVE-1) of in vivo Matrigel plugs revealed that plugs were well vascularized for the MeWo cells (Figure 5A(b,c)), A375 cells (Figure 5B(h,i)), and SK-MEL-28 cells (Figure 5C(n,o)), suggesting the capacity of the melanoma cells to recruit vascular and lymphatic endothelial cells to develop and intensify angiogenesis and lymphangiogenesis, respectively. In contrast, αAMR treatment induced a clear decrease in angiogenesis and lymphangiogenesis in the plugs injected with MeWo cells (Figure 5A(e,f)), A375 cells (Figure 5B(k,l)), and SK-MEL-28 cells (Figure 5C(q,r)). Quantification of CD31-positive endothelial cells and LYVE-1 positive lymphatic cells demonstrated a marked decrease in the number of both cell types in plugs from animals treated with αAMRs compared to animals treated with control-IgG (*p* < 0.01; *p* < 0.001; Figure 5D–F) These data strongly suggest that a part of the angiogenesis and lymphangiogenesis revealed in plugs is due to AM secreted by melanoma cells.

**Figure 6:**
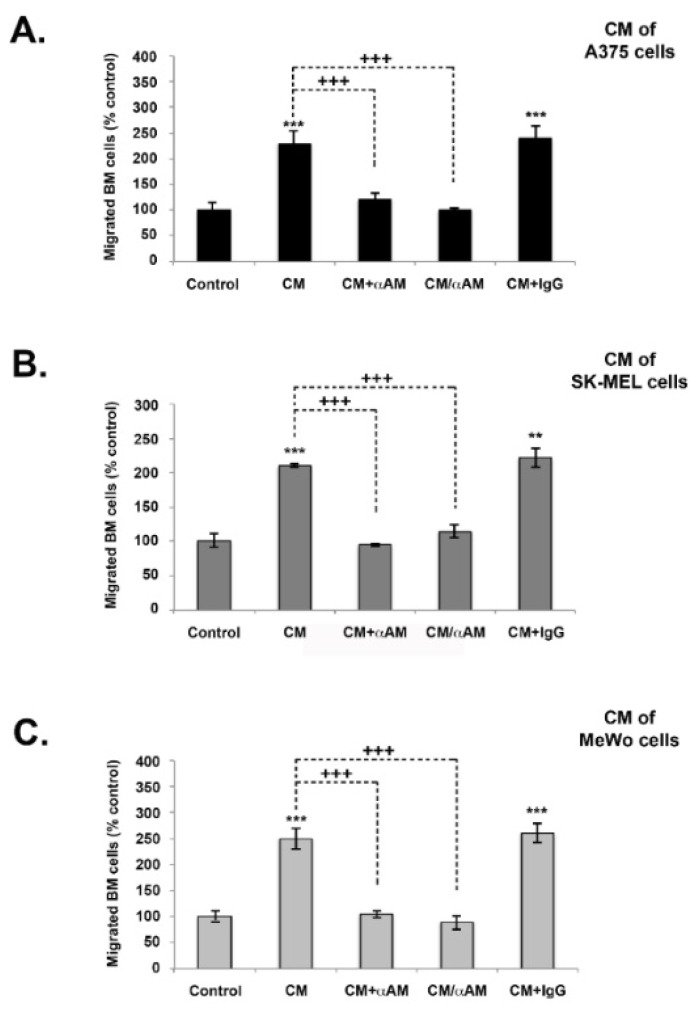
The effect of in vitro melanoma cells-conditioned medium (CM) induced invasion of BMDCs in the AM signaling blockade. (**A**–**C**) In vitro regulation of BMDCs by A375, SK-MEL-28, and MeWo cells-CM. For all chambers, the bottom well was filled with melanoma cells-CM, while the control well was filled with DMEM containing 2% FBS (control). Immunoreactive AM secreted in CM was neutralized with pretreatment with αAM (70 μg/mL) for 30 min. αAMRs (70 μg/mL) or control IgG (70 μg/mL) was used to pre-treat bone marrow cells (5 × 10^5^ cells), which were then placed in the upper chamber and incubated (see Materials and Methods). Migrated cells were stained with DAPI and counted under the microscope at 50× magnification. Numbers represent the number of migrated cells in 10 high-power fields, given as means ± SD of four independent experiments, each performed in triplicate. The asterisk (*) is used for comparison to control cells (** *p* < 0.01; *** *p* < 0.001) and the plus symbol (+) is used for comparison to CM-treated cells (+++ *p* < 0.001).

We additionally conducted tests to assess whether AM secreted by melanoma cells was implicated in the recruitment of endothelial-like cells and pericytes-like cells in a functional and stable angiogenic process. Previous research has demonstrated, using in vivo Matrigel plug bioassays, that AM can induce recruitment of AMR^+^ cells, including macrophages/monocytes, pericytes, and endothelial-like cells [21]. Analysis of invasion assays reveals that melanoma cells-conditioned medium (A375-CM, SK-MEL-28-CM, and MeWo-CM) promoted invasion of cells derived from bone marrow (BMDCs) from the femurs of C57BL/6 mice in a transwell assay (Figure 6A–C). The stimulating effects of melanoma cells-CM on invasion were significantly inhibited by application of function-blocking AM antibodies (αAM), or by pre-incubation with αAMRs (Figure 6A–C). Pre-incubation with control-IgG had no effect on invasion (Figure 6A–C). These results strongly support the hypothesis that the AM system is a factor responsible for the involvement of endothelial-like cells, BMDCs, and pericytes/smooth-muscle cells in the promotion of stable and functional lymphangiogenesis and angiogenesis.

### 3.7. AM Blockade Inhibits the Growth of MeWo Tumor Xenografts In Vivo

**Figure 7:**
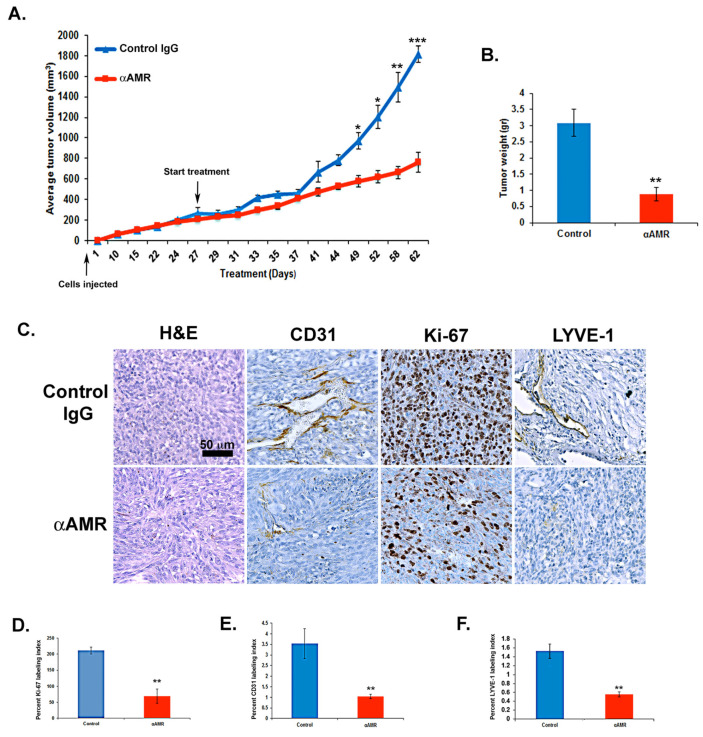
AM signaling blockade inhibited the growth of MeWo xenografts in vivo. (**A**) MeWo cells (2 × 10^6^) were injected subcutaneously into the flanks of athymic nude mice (6 weeks old) (n = 10 in each group). Mice with tumor volumes averaging 250 ± 50 mm^3^ received intraperitoneal injections of αAMRs (12 mg/kg) every 3 days. Control mice were treated with 12 mg/kg of nonspecific isotype control immunoglobulin G (IgG). Measurements of tumor volume demonstrate differences in the growth of animals treated with αAMRs (n = 10) and control IgG (n = 10) during the 52-day schedule, * *p* < 0.05; ** *p* < 0.01; *** *p* < 0.001. (**B**) Tumors were weighed immediately after excision and the average tumor is indicated as the mean ± SD (n = 10), ** *p* < 0.01. (**C**) αAMRs-treated tumors are less vascular and depleted of vascular and lymphatic endothelial cells. LYVE-1, CD31, and Ki-67 antibodies and hematoxylin and eosin were used to stain the tumor sections. The figure depicts Ki-67 positive cells, with each section analyzed using 10 magnification fields (400×). Microvessel density was determined using immunohistochemical staining of the CD-31 marker of the endothelial cell surface. The density of cells staining positive for Ki-67 (**D**), CD-31 (**E**), or LYVE-1 (**F**) was assessed quantitatively based on the entire slide surface using CALOPIX Software v2.10.16 by Tribvn. Analysis was conducted with MVF_Image J1.52a software. The values shown represent the means ± SD, ** *p* < 0.01.

The role of AM in melanoma tumorigenesis was assessed by examining the impact of inhibiting AM signaling on tumor xenografts. Athymic nude mice with established MeWo xenografts (>200 mm^3^) were treated with either αAMRs (treatment group) or a rabbit IgG (control group). Treatments were administered every three days by i.p. injection (12 mg/kg for both). Tumor volume was measured throughout the study period as a measure of growth. Results indicated that xenograft growth was significantly inhibited by treatment with αAMRs compared to control (Figure 7A). After 25 days of treatment had elapsed (52 days after initial cell injection), five animals were humanely killed for assessment of tumor size and vascularity. Mean tumor weights for mice given control IgG and αAMRs treatments were 3 ± 0.8 g and 0.9 ± 0.25 g, respectively, after 25 days of treatment (Figure 7B). Tumors from mice treated with αAMRs were pale and showed clearly diminished vasculature, whereas tumors in the control group were found to be larger with extensive vascularization (not shown). Of note, three mice in the control group showed the development of liver metastasis; meanwhile, no metastasis could be found in αAMRs-treated animals.

### 3.8. AM Blockade Decreases Tumor Cell Proliferation and Impairs Tumor Angiogenesis and Lymphangiogenesis In Vivo

There was a significant difference in the Ki-67 labeling index between mice treated with αAMRs antibodies and control IgG (*p* < 0.001; Figure 7C,D), as demonstrated by immunohistochemical staining of tumor xenografts. This analysis also showed that tumor vascularization was deeply disrupted among mice treated with αAMRs, consistent with the hypothesis that AM signaling inhibition would decrease lymphangiogenesis and angiogenesis (Figure 7C). There was also a clear decrease in both CD31-positive endothelial cells and LYVE-1-positive lymphatic endothelial cells, further demonstrating a decrease in both cell types in treated mice compared to controls (*p* < 0.01) (Figure 7E,F).

## 4. Discussion

A better understanding of early migration and invasion in primary tumor disease is crucial for building prognostic and predictive models and improving adjuvant strategies in early tumors, and a better understanding of the microenvironment and progression/migration in the metastatic setting is paramount for improving the efficacy of metastatic treatments. Among the multiple factors involved in tumor development, autocrine and paracrine factors delivered in the microenvironment may play an important role via their effect on blood and lymphatic vascularization. Identifying factors produced in the tumor and elucidating their roles in tumor development may provide clues for improving therapy.

The purpose of this study was to examine how AM and its receptors are expressed in primary and metastatic melanoma in order to shed light on its potential role as an autocrine/paracrine growth factor. This was assessed in both in vitro and in vivo studies. Immunohistochemical analysis was employed to reveal that AM and AM receptors are localized primarily in melanoma cells. This pattern of expression provides strong evidence that the AM system may be involved in melanoma progression. Previous research demonstrated similar patterns of AM localization in serial sections among specimens of kidney cancer, colorectal cancer, prostate cancer, and epithelial mesothelioma [14,22,29,30].

The link between hypoxia and melanoma is well established [31]. The results of this study support these findings, demonstrating that AM expression in A375, SK-MEL-28, and MeWo cells increased by a substantial magnitude under hypoxic conditions. These findings suggest the possibility that a similar increase may occur under hypoxic conditions in the tumor microenvironment (e.g., immune, proangiogenic, and provascular cells). Previous research has revealed that a major source of AM in melanoma is tumor-associated macrophages (TAM) [13]. As AM is produced and secreted in hypoxic regions of tumors [32,33], this may contribute to tumor growth resulting from autocrine/paracrine-mediated proliferation. Furthermore, AM is known to possess angiogenic and vasodilator functions [7,34], which may account for its role in facilitating nutritional supplementation with tumor cells, as well as its functionality in the formation of a stable vascular network. Finally, AM serves to reduce the rate of cellular apoptosis [35], which may have the impact of rescuing tumor cells selectively from cell death, thus leading to the development of tumors with a more malignant phenotype [32].

This study confirms that AM and the AM receptors AM_1_ and AM_2_ are present in melanoma tissue, as previously reported by Martinez et al. [12] and Chen et al. [13], which supports the view that AM may play a role as an autocrine/paracrine growth factor in melanoma. Specifically, we found that AM and its receptors were expressed in A375, SK-MEL-28, and MeWo melanoma cells, and furthermore that treatment with αAM and αAMR inhibited proliferation of A375 and SK-MEL-28 cells. Conversely, although the AM system was expressed in MeWo cells, there was no inhibitory treatment effect on the proliferation of these cells in vitro. This negative finding is likely to be due to the loss of NF1 in MeWo cells, which has been found to be associated with MEK dependence and RAS activation [36]. However, the present results demonstrated that AM induced migration and invasion in vitro in all three melanoma cell lines. This pattern of results (except for the negative finding regarding inhibition of proliferation in MeWo cells) is consistent with the hypothesized autocrine loop linking the AM system with tumor dynamics in melanomas. Within tumors, AM-producing cells may act to stimulate AM receptor-expressing cells via autocrine/paracrine mechanisms. Our findings are consistent with those reported previously regarding other cancer models [10,11].

Previous research supports multiple mechanisms linking AM with malignancy, including inhibition of apoptosis, stimulation of tumor cell proliferation, stabilization of angiogenesis, and shortened doubling-time in several different types of cancer [20,21,22,23,24,25,26,30]; the same mechanisms may also be relevant in the development of melanoma. We demonstrated that the addition of melanoma cells (A375, SK-MEL-28, and MeWo) to Matrigel plug bioassays in vivo significantly enhanced plug neovascularization, which was effectively inhibited by systemic injection of αAMRs. These data indicate that AM produced by melanoma cells can recruit circulating AMR^+^ cells into in vivo Matrigel bioassays to construct a neovascular network supported by the invasion of BMDCs in vitro, which is induced by AM secreted in conditioned medium from melanoma cells. Previously, we showed the capability of AM to recruit and entrap diverse proangiogenic cells (CD45^+^ cells, MOMA^+^ cells, etc.) and provascular cells (endothelial-like cells, α-SMA^+^ mural cells) to promote angiogenesis in in vivo Matrigel bioassays [21]. Consistent with the findings of previous research, we have found that AM can act to induce neovascularization and vessel stabilization [3,21,37].

In order to examine the effect of αAMR therapy on tumor growth via angiogenesis, we used MeWo cells since their own proliferation is not directly inhibited by AM blockade in vitro, which might be due to the loss of NF1 activity by these cells [36]. Our data demonstrate that αAMR can be delivered efficiently in vivo and significantly suppresses growth in established MeWo xenografts, which may be attributable to the creation of a hostile microenvironment within the tumor. Specifically, anti-CD31 antibody immunostaining showed that this treatment resulted in the disappearance of more than 80% of vessels and clear depletion of endothelial cells, while vessel density in lumens also showed a substantial decrease. Collectively, these findings provide strong support for the hypothesis that the AM system is implicated in the vessel stabilization and/or neovascularization processes in melanoma. Microvessel loss in the tumors treated with αAMRs may indicate that stimulation of the CLR/RAMP2/RAMP3-expressing tumor vasculature by AM is a survival mechanism rooted in the proliferation of tumor endothelia. Similar findings have also been reported regarding the A549 lung cancer cell line, which demonstrated no inhibition of proliferation in vitro in response to the AM system being blocked because of the KRAS^G12S^ mutation leading to permanent activation of the MAP kinase pathway [21]. Crucially, treatment with αAMRs does not disrupt physiological vascularization in normal tissue, which has a much longer doubling-time of approximately 3 years, suggesting a highly active role for the AM system in tumor neoangiogenesis with its doubling-time of only a few days [38]. Our findings agree with the data reported by Chen et al. in which they found that tumor growth was significantly inhibited by the AM receptor antagonist, AMA, in both B16/F10 mouse and A375 human melanoma models [13]. Collectively, these observations suggest that AM produced by tumor-associated macrophages (TAM), probably induced by hypoxia in the tumor environment and melanoma cells as reported in this study, is a key factor in inducing angiogenesis and melanoma growth.

Another critical element in tumor pathogenesis is lymphangiogenesis [39,40,41]. In both human tumors and animal models, the risk of metastasis is increased by intra- and/or peritumoral lymphangiogenesis [39]. In the present study, we examined whether αAMRs treatment resulted in impairment of lymphatic vessels in MeWo xenografts and in vivo Matrigel bioassays by analyzing these vessels with the murine LYVE-1 antibody. We found that tumors treated with αAMRs were completely free of these vessels. By contrast, inhibition treatment did not have an impact on mature lymphatics, which suggests that whereas AM receptor activation is a necessary component of lymphatic growth, it is not an element of ongoing lymphatic maintenance. In previous studies, we reported that AM receptor activation induces proliferation, migration, invasion, and survival of LECs [22], which likewise suggests that AM plays a key role in lymphatic vessel development during tumor growth. Thus, these results together support the hypothesis that the activity of AM and its receptors upon the induction of AM expression [11,27,42] impacts tumor formation by promoting lymphangiogenesis, in addition to its impact on neoangiogenesis and on tumor cell growth. In another previous study, we demonstrated the impact of the AM system on lymphangiogenesis in prostate cancer using Du145 xenografts [22]. Other research has found that loss of the *AM*, *CALCRL*, or *RAMP2* genes results in the reduced proliferation of LECs in jugular lymphatic vessels [43]. Additionally, it has been found that administering AM can stimulate both angiogenesis and lymphangiogenesis in the lymphatic vessels at the site of injury in mice [44]. It has been well established that there are two ways in which tumor cells may enter the lymphatic vasculature, either by invasion of existing lymphatic vessels peripheral to the tumor, or by inducing lymphangiogenesis through the production of growth factors [45,46]. A possible conjecture based on these observations is that AM produced by tumor cells may facilitate the entry of tumor cells into the lymphatic endothelium by stimulating the growth and dilation of peritumoral lymphatic vessels, also preventing increases in tumor pressure, as identified in previous findings for other lymphangiogenic growth factors [47,48], VEGF [49,50], and platelet-derived growth factor-BB (PDGF-BB) [51].

Of note, no metastasis could be found in αAMRs-treated animals. The finding reported by Tanaka et al. [52] indicates that the deletion of RAMP2 from endothelial cells suppresses the growth of locally transplanted B16F10 melanoma cells. Spontaneous lung metastasis was analyzed using B16BL6 established from B16F10 melanoma cells and showed that the incidence of metastasis and the number of metastatic lesions were higher in drug-inducible endothelial cell-specific RAMP2 knockout mice (DI-E-RAMP2^−/−^), which could be due to the endothelial cells that were deformed and facilitating the infiltration of the inflammatory cells of the vessel walls. The inflammatory cells could express the chemotactic factors S100A8/9 and SSA3, which attract tumor cells and mediate the formation of a pre-metastatic niche [52]; this is contrary to the present study where no metastasis could be found in all the animals treated with αAMR, while contrastingly three animals in the control group did show metastasis in the liver. Different points of view could explain this discrepancy. First, endothelial cells participating in neovessel assembly are in a dynamic state during tumor angiogenesis and are thus not firmly attached to the extracellular matrix or to peri-endothelial cells such as pericytes. AM blockade using αAMR exerts an anti-vascular and anti-angiogenic effect by presumably taking advantage of the relative instability of tumor vasculature and its supporting structures, thereby inducing endothelial cell death [53] and a collapse and regression of tumor vascular and lymphatic neovessels, which are the routes used by tumor cells for metastasis. Second, contrary to DI-E-RAMP2^−/−^ that could affect most of the endothelial cells in the animal, as in the lung for example, the AM system blockade using αAMR did not disrupt the normal vasculature of different organs in animals bearing tumors, such as in the kidney [53], prostate [22], lung, and liver (unpublished data), probably because of the AM system that is expressed at the very low level in non-angiogenic endothelium that cannot be detected by αAMR in vivo. Intravenous injection of fluorescent αAM into animals bearing U87 xenografts in the brain localized specifically at the tumor site 24 h later, without any fixation in the rest of the body (unpublished data). Third, it is also possible that αAMR treatment could prevent any growth of metastatic niches by inhibiting vascular neoangiogenesis and lymphangiogenesis, thus impairing any growth at the secondary tumor site.

Our data on melanoma suggest that a blockade of the AM system might help to constitute a hurdle in the metastatic process toward lymph nodes and other organs via an impediment to neoangiogenesis and lymphangiogenesis. Efforts are underway to develop mono-specific and/or bi-specific mAbs targeting AM/AM receptors, and the development of a small-molecule AM antagonist is also being explored [11,54,55].

## 5. Conclusions

AM is a gene that is highly expressed in tumors induced by hypoxia caused either by tumor growth and/or post-therapeutic treatment. This study showed that AM stimulates melanoma cell proliferation, migration, and invasion in vitro. The in vivo study highlights its strong contribution to neoplastic angiogenesis and lymphangiogenesis. AM system blockade inhibits melanoma cell growth, migration, and invasion in vitro and tumor growth in vivo by disrupting neoangiogenesis and lymphangiogenesis. Taken together, these results confirm that the AM system could be used as a target to treat melanoma and to prevent metastasis.

## Data Availability

Not applicable.

## Supplementary Materials

The following supporting information can be downloaded at: https://www.mdpi.com/article/10.3390/cancers14235909/s1, Figure S1: Expression of AM and its receptors in human melanoma. Immunohistochemistry of melanoma tissues with antiserum preincubated with human synthetic AM, CLR, RAMP2, and RAMP3 at 50 μM each is shown. No staining for AM, CLR, RAMP2, and RAMP3 can be observed suggesting the specificity of the staining reported in Figure 1; Figure S2: Expression of AMR (CLR, RAMP2, and RAMP3) in melanoma cell lines. Total RNA (1 μg, DNA free) prepared from MeWo, SK-MEL-28 and A375 cells were reverse transcribed into cDNA under normoxia and hypoxia conditions. Relative human CLR (A), RAMP2 (B), RAMP3 (C) and GAPDH mRNAs levels were amplified, detected, and quantified in real time by using an LC480 polymerase chain reaction (PCR) system (Roche Diagnostics, Meylan, France) as described previously (Berenguer et al., [27]). No significant differences between cells treated with hypoxia mimetic DFX and untreated control cells in CLR, RAMP2, and RAMP3 expression. Each experiment is representative of five independent experiments.
