# Supplementary material for: Adrenomedullin Secreted by Melanoma Cells Promotes Melanoma Tumor Growth through Angiogenesis and Lymphangiogenesis
Source: Cancers (Basel). 2022 Nov 29;14(23):5909. doi: 10.3390/cancers14235909 (PMC9738606; doi:10.3390/cancers14235909)
Supplement: Supplementary file 1 [file cancers-14-05909-s001.zip › cancers-2026864-supplementary.pdf]

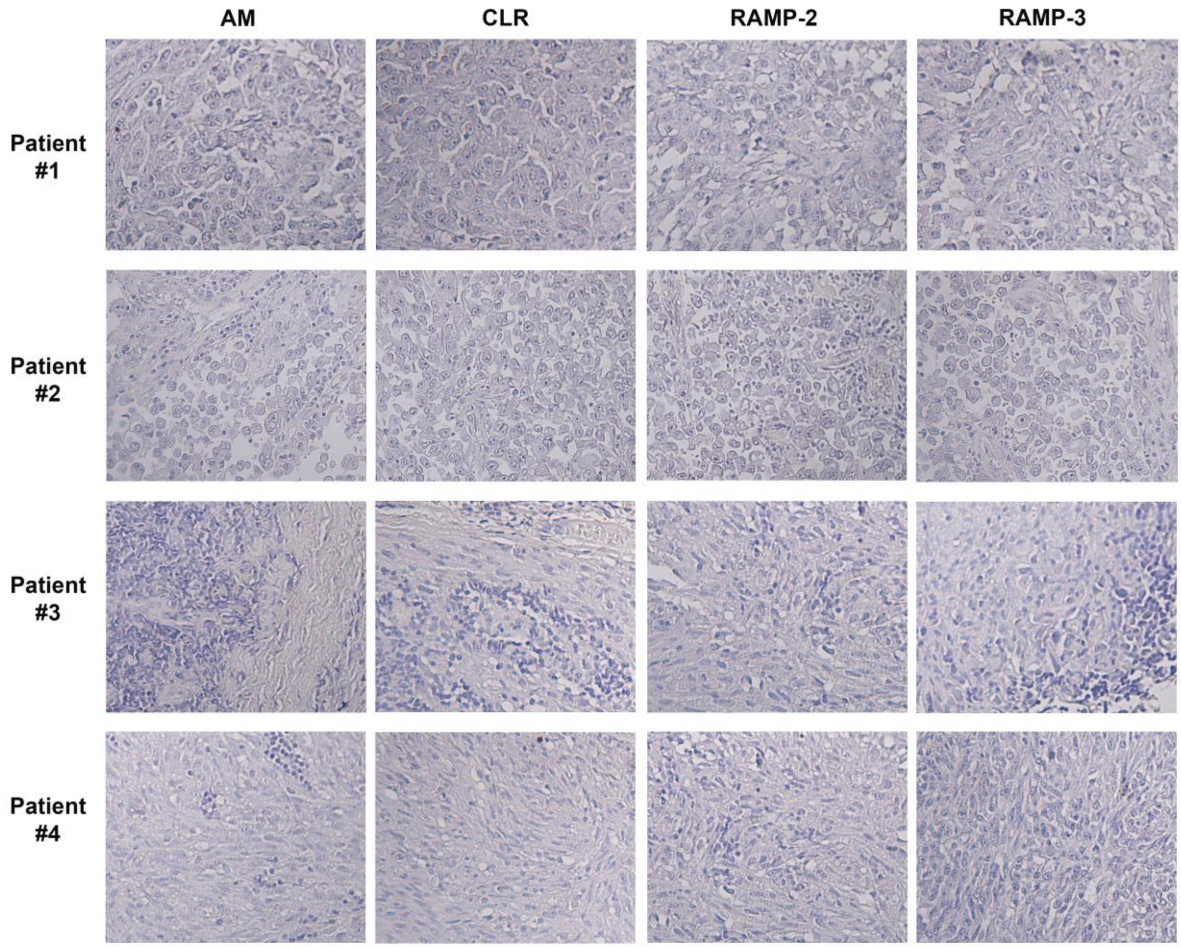

Supplementary Figure S1. Expression of AM and its receptors in human melanoma. Immunohistochemistry of melanoma tissues with antiserum preincubated with human synthetic AM, CLR, RAMP2, and RAMP3 at 50 μM each is shown. No staining for AM, CLR, RAMP2, and RAMP3 can be observed suggesting the specificity of the staining reported in Figure 1.

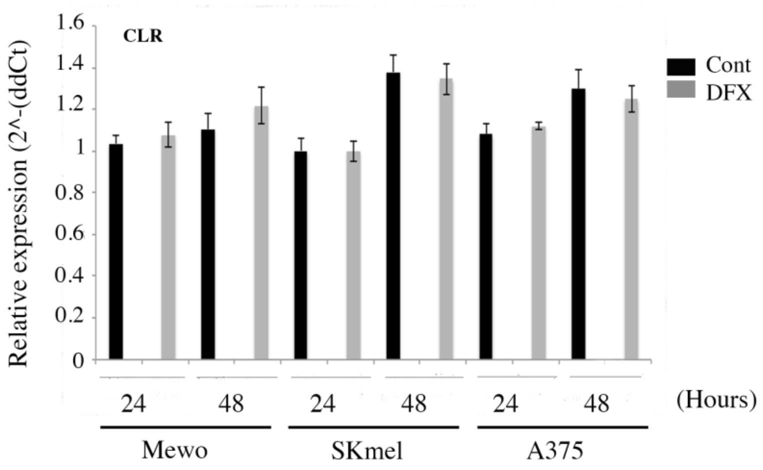

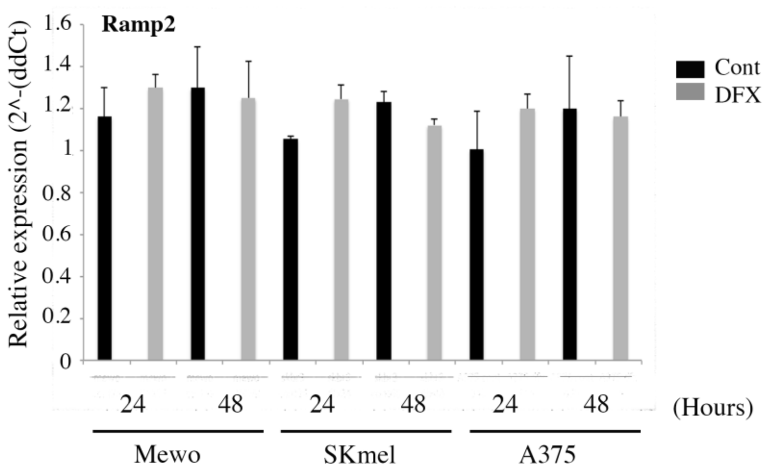

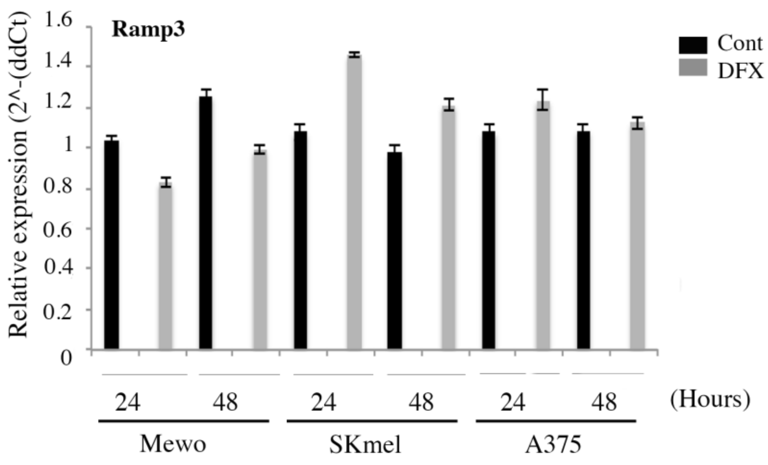

Supplementary Figure S2: Expression of AMR (CLR, RAMP2, and RAMP3) in melanoma cell lines. Total RNA (1µg, DNA free) prepared from MeWo, SK-MEL-28 and A375 cells were reverse transcribed into cDNA under normoxia and hypoxia conditions. Relative human CLR (**A**), RAMP2 (**B**), RAMP3 (**C**) and GAPDH mRNAs levels were amplified, detected, and quantified in real time by using an LC480 polymerase chain reaction (PCR) system (Roche Diagnostics, Meylan, France) as described previously (Berenguer et al., [27]). No significant differences between cells treated with hypoxia mimetic DFX and untreated control cells in CLR, RAMP2, and RAMP3 expression. Each experiment is representative of five independent experiments.
